# E-cigarettes use among university students in Jordan: Perception and related knowledge

**DOI:** 10.1371/journal.pone.0262090

**Published:** 2021-12-31

**Authors:** Nour A. Al-Sawalha, Basima A. Almomani, Enas Mokhemer, Samah F. Al-Shatnawi, Roba Bdeir

**Affiliations:** 1 Department of Clinical Pharmacy, Faculty of Pharmacy, Jordan University of Science and Technology, Irbid, Jordan; 2 Faculty of Pharmacy, Jadara University, Irbid, Jordan; International Medical University, MALAYSIA

## Abstract

The use of e-cigarettes has been increasing in popularity among people, especially young adults. Assessing young individuals’ perceptions of e-cigarettes can help to identify factors that may influence their decision to use e-cigarettes. To examine prevalence, perceptions, and knowledge of e-cigarettes among university students in Jordan, an observational cross-sectional study using an online self-administered questionnaire was conducted among students from public and private universities between October 2020 and January 2021. A total of 1259 university students completed the questionnaire. Approximately, 11% of participants reported e-cigarettes use. Among users, 26.5% used it for the purpose of smoking cessation, while 22% of them used it out of curiosity, and 20.5% used it as they believed it is less harmful than other tobacco products. Multivariate analysis showed that conventional cigarette smokers were independently associated with a better knowledge about e-cigarettes (OR = 1.496, 95CI% = 1.018–2.197, p-value = 0.040). In addition, medical students showed a significantly better knowledge compared to non-medical students (OR = 1.710, 95CI% = 1.326–2.204, p-value = <0.001). In Jordan, e-cigarettes use is less popular compared to other countries. Nonetheless, educational interventions are needed to correct misconceptions about e-cigarettes among young adults.

## Introduction

The use of electronic cigarettes (e-cigarettes) has grown considerably especially among adults and younger adults [1, 2]. According to the Center for Disease Control and Prevention (CDC), 8.1 million U.S. adults used e-cigarettes in 2018, with the highest prevalence among individuals in the age range of 18–24 years [2]. A study conducted among university students in Saudi Arabia reported a percentage of 27.7% of e-cigarette users, which is almost the double of conventional cigarette smoking percentage [3].

E-cigarettes are devices that are designed to heat a solution composed of humectants (glycerol or propylene glycol), nicotine, and in many cases, flavoring agents to deliver the aerosol to be inhaled by users [4–6]. Analysis of e-cigarettes’ vapor revealed the presence of harmful substances such as formaldehyde, acetaldehyde and acrolein [7–9]. Formaldehyde and acetaldehyde are classified as possibly carcinogenic substances while acrolein causes lung injury as well as nasal irritation [7, 9]. It has been shown that the use of e-cigarettes is not risk free. E-cigarettes use induce airway inflammation, alveolar injury, ciliary dysfunction, and increase mucus secretion [10]. Users of e-cigarette had higher risk to develop acute health conditions such as stroke, myocardial infarction and coronary artery diseases compared to nonsmokers [11]. Furthermore, evidence of a higher addictive potential with e-cigarettes than conventional cigarettes among young adults was presented [12, 13].

Advertising greatly affects young adults; e-cigarettes are marketed as an effective approach for reducing conventional cigarette consumption and smoking cessation [14, 15]. Marketing messages claim that e-cigarettes are safer, cleaner and can be used in places where conventional smoking is restricted [5, 16].

As its popularity and use increases, so is the concern about public health [17]. Therefore, public awareness about the harmful effects of e-cigarettes has been examined. A cross-sectional study among adults in USA showed that about 78% of respondents were aware of at least one harmful effect of nicotine [18]. Gupta and colleagues reported low awareness level of the side effects related to e-cigarette use [19]. Further, around 63% of Lebanese participants shown low level of knowledge about e-cigarettes [20]. Almutham and colleagues presented that even medical students had low level of knowledge about e-cigarettes competitively [21].

As the use of e-cigarettes continues to increase along with uncertainty about its safety, it is of paramount importance to explore the patterns, perceptions, and attitudes of e-cigarette smokers. In Jordan, recent studies have reported prevalence of e-cigarettes use ranging between 11.7–18% [22, 23]. The latest study on the knowledge of e-cigarette reported its finding among adults within the Jordanian community [24]. The prevalence of smokers and dual smokers were 11.7% and 4.0%, respectively, and poor knowledge about the content and types of e-cigarettes was reported. More than two third of the participants thought vaping cannot be addictive nor can it be harmful to children and pregnant women. These studies provided important insights to the knowledge and beliefs toward e-cigarettes among the Jordanian adult community. However, there are limited information about perceptions and knowledge of the harmful effects of e-cigarettes within the younger adults, especially among university students in Jordan. Thus far, all the studies investigating e-cigarettes within Jordan focused on the older adult population. Assessing young individuals’ perceptions of e-cigarettes can help to identify factors that may influence their decision to e-cigarettes use. In addition, it has the potential to implement effective regulations and educational programs that particularly address e-cigarettes use. This study examined the perceptions, and knowledge of e-cigarettes among university students in Jordan.

## Methods

### Study population and design

A cross-sectional questionnaire-based study was conducted among undergraduate and graduate students in Jordan across different geographical areas, North, Middle and South Jordan, between October 2020—January 2021. Inclusion criteria include being attending private and public universities and have access to internet. Students who were not willing to participate in the study were excluded. University students were directly recruited to participate through their official students’ groups pages from all Jordanian universities on social media portals such as Facebook^®^.

The ethical approval was granted from institutional review board (IRB) at Jordan University of Science and Technology (JUST) (approval number: 48/134/2020).

### Study instrument

Survey items were adopted from previously conducted studies [25–28]. Then, some items were modified based on discussions among researchers. The instrument in English language was reviewed by an expert panel from the field for face validity, then it was translated and examined for clarity through a pilot study (n = 20). The internal consistency of the questionnaire measured by Cronbach’s alpha ranged from 0.690 (perception section) to 0.795 (knowledge section) which indicated good reliability. The questionnaire comprised four main sections: demographics, smoking habits, perceptions, and knowledge. Smoking habits consisted of six items with closed ended answers. The perception section composed of five items with three answer options (agree, neutral, disagree). As compared to conventional smoking, perception items were added to assess perceived health and behavioral outcome expectations related to e-cigarette use. The knowledge part comprised 11 items with three answer options (true, false, do not know). The estimated time to complete the survey was 5–7 minutes.

### Statistical analysis

All collected data were entered to Statistical Package for Social Sciences (SPSS, version 23). Descriptive analysis was used to present the data as counts (percentage). The knowledge score (out of 11) was dichotomized based on cutoff point into: knowledgeable (≥7) and non-knowledgeable (<7) participants. For analysis, the answer of “do not know” was considered as “incorrect” answer. Univariate analysis was performed using Chi square test and variables with p value less than 0.25 were entered in multivariate analysis. Independent factors affecting students’ knowledge was determined using binary logistic regression and both odds ratio (OR) and 95% confidence interval (95%CI) were calculated. P-value less than 0.05 was considered statistically significant.

## Results

### Demographics

A total of 1259 university students were included in this study. More than half of them (54.9%) aged 18–20 years old and two thirds were females (70%). Most of participants (83.2%) studied in public universities, 92.2% were registered in undergraduate programs and 63.7% were enrolled in medical colleges. All demographic details are shown in Table 1. The sources of information about e-cigarettes use were examined. Social media (43.1%) was the most common source followed by friends (32.9%) and families (13%). However, only 2.6% of participants did not hear about e-cigarettes before (S1 Fig).

**Table 1:** Demographics and characteristics of participants.

Characteristic	N (%)
**Age**	
18–20 year	688 (54.9)
21–23 year	394 (31.4)
≥24 year	172 (13.7)
**Gender**	
Female	880 (70.2)
Male	373 (29.8)
**University**	
Government	1041 (83.2)
Private	210 (16.8)
**Residency**	
North	451 (37.2)
South	252 (20.8)
Middle	509 (42.0)
**Educational program**	
Undergraduate	1140 (92.2)
Postgraduate	97 (7.8)
**Study year**	
1^st^ & 2^nd^	579 (46.7)
3^rd^ & 4^th^	473 (38.1)
5^th^ & 6^th^	188 (15.2)
**College**	
Medical	794 (63.7)
Non-medical	452 (36.3)

### Smoking habits among participants

Only 11.3% of students were current conventional cigarette smokers, while 10.5% were e-cigarettes smokers. Ex-smokers are individuals who did not smoke at the time of the interview, but they smoked cigarettes regularly before. More than half of e-cigarettes smokers (55.7%) had been smoking them for 1–3 years. Among e-cigarettes smokers, 59.8% smoked them on daily basis and 62.9% of them spent 30 minutes and more in each smoking session (Table 2). Participants reported different reasons to using e-cigarettes for the first time such as to stop conventional cigarette smoking (26.5%), curiosity (22%) and their belief that e-cigarettes are safer than conventional cigarette smoking (20.5%) (S2 Fig).

**Table 2:** Smoking habits of participants.

Smoking habits of participants	N (%)
**Do you smoke conventional cigarettes?**	
No	1047 (83.5)
Yes	142 (11.3)
ex-smoker	65 (5.2)
**Do you smoke e-cigarettes?**	
No	1083 (86)
Yes	132 (10.5)
ex-smoker	44 (3.5)
**Since when do you smoke e-cigarettes?**	
< one year	43 (32.8)
1–3 year	73 (55.7)
>3 year	15 (11.5)
**How many times do you smoke e-cigarettes?**	
Daily	79 (59.8)
Weekly	35 (26.5)
Monthly	18 (13.6)
**How many minutes do you smoke e-cigarettes?**	
< 30 min	49 (37.1)
30–60 min	41 (31.1)
>60 min	42 (31.8)

### Perceptions of university students about e-cigarettes

**Fig 1:**
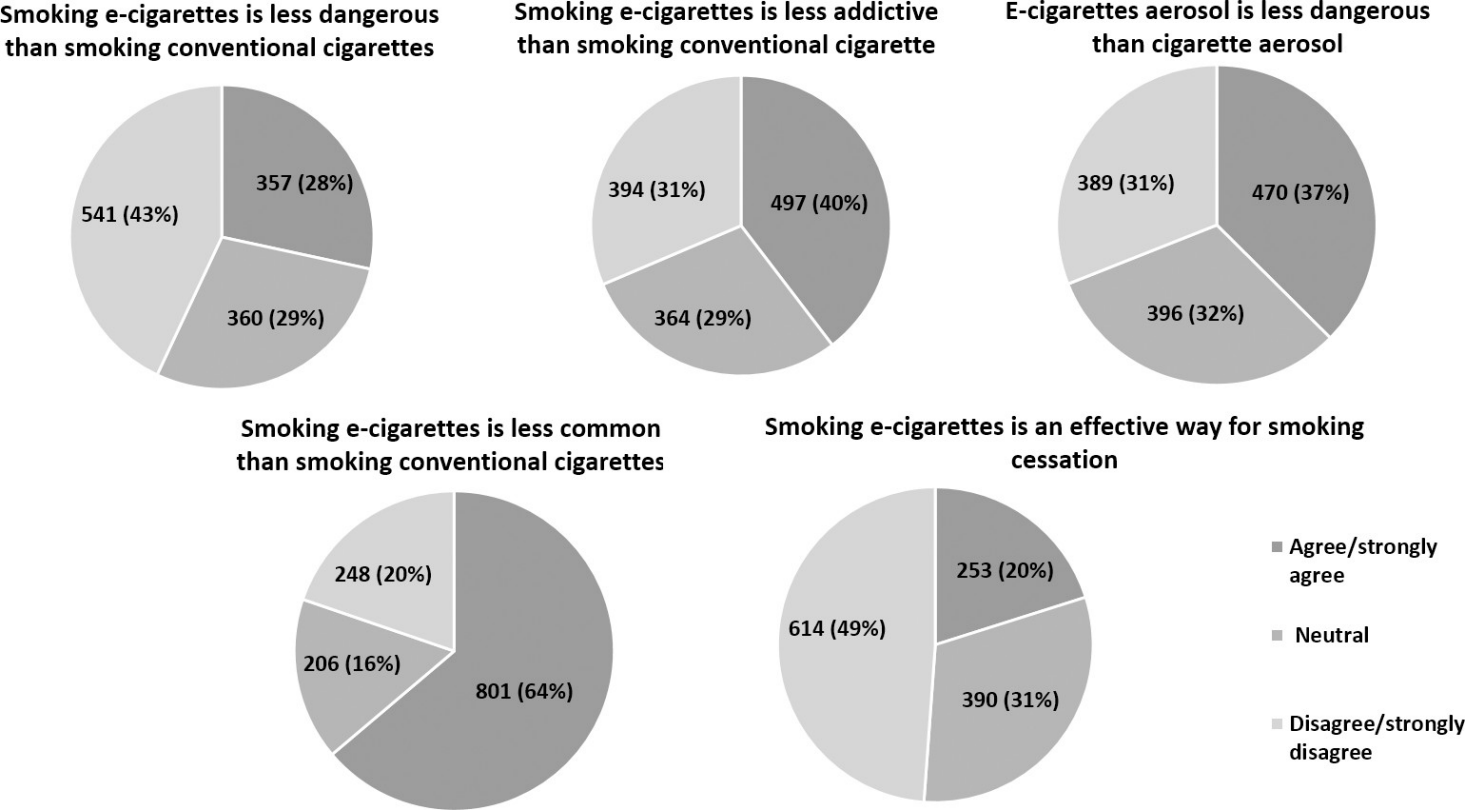
Perception of participants about e-cigarettes.

Approximately 30% agreed that smoking e-cigarettes is less dangerous than conventional cigarette smoking. More than a third (40%) agreed that smoking e-cigarettes is less addictive than conventional cigarette smoking. Most of participants (63.8%) reported that smoking e-cigarettes is less common than conventional cigarette smoking. Only 20% thought that smoking e-cigarettes is an effective way for cessation of conventional smoking. When asked about the probability of advising friends or family members to smoke e-cigarettes instead of conventional cigarettes, 27.2% reported “yes”, 61.9% reported “no”, and 10.9% were “neutral”. Fig 1 summarizes participants’ perceptions toward e-cigarettes use.

Current and ex-smokers, compared to non-smokers, significantly perceived e-cigarettes use as less dangerous, less addictive and as an effective way for cessation of conventional smoking (P<0.001). Table 3 summarizes perceptions of participants by their smoking status.

**Table 3:** Perception of participants about e-cigarettes by e-cigarette smoking status (N = 1259).

Perception	E-cigarette smoking status N (%)		P value
	No	Yes/ex-smoker	
**Smoking e-cigarettes is less dangerous than smoking conventional cigarettes**			<0.001
Agree	269 (24.8)	88 (50.3)	
Disagree/neutral	814 (75.2)	87 (49.7)	
**Smoking e-cigarettes is less addictive than smoking conventional cigarette**			<0.001
Agree	398 (36.9)	99 (56.3)	
Disagree/neutral	681 (63.1)	77 (43.8)	
**E-cigarettes aerosol is less dangerous than cigarette aerosol**			<0.001
Agree	373 (34.5)	97 (55.4)	
Disagree/neutral	707 (65.5)	78 (44.6)	
**Smoking e-cigarettes is less common than smoking conventional cigarettes**			0.055
Agree	700 (64.9)	101 (57.4)	
Disagree/neutral	379 (35.1)	75 (42.6)	
**Smoking e-cigarettes is an effective way for smoking cessation**			<0.001
Agree	173 (16)	80 (45.5)	
Disagree/neutral	908 (84)	96 (54.5)	

### University students’ knowledge about e-cigarettes

Average score of knowledge was 5.26± 2.86 (mean± standard deviation) (out of 11). Reported scores ranged between zero and 11. Less than half of participants (44.0%) knew that e-cigarettes are reusable. In addition, approximately 60% of them had the misconception that all e-cigarettes contain natural substances, and 32.1% of them knew that e-cigarettes are source of second-hand exposure to nicotine. Furthermore, 57.2% of participants knew that e-cigarettes contain carcinogenic ingredients. Most of them knew that e-cigarettes’ aerosol increases the heart rate/ arterial stiffness (58.5%), blood pressure (57.7%) and induces obstruction of conducting airways (~70%).

Age, gender, program, study year, current conventional cigarette and e-cigarettes smoking status were factors that were investigated as predictors for students’ knowledge about e-cigarettes. The multivariate analysis showed that college and conventional cigarette smokers were independently associated with knowledge about smoking e-cigarettes (Table 4). Students from medical colleges were more knowledgeable about e-cigarettes than nonmedical college students (OR = 1.710, 95CI% = 1.326–2.204, p value<0.001). In addition, conventional cigarette smokers were more knowledgeable about e-cigarettes than those who were not (OR = 1.496, 95CI% = 1.018–2.197, p value = 0.040).

**Table 4:** Predictors of knowledge about smoking e-cigarettes.

Factors	OR (95%CI)	P value
**Gender**		0.132
Female	Ref	
Male	1.238 (0.938–1.634)	
**College**		<0.001
Non-Medical	Ref	
Medical	1.710 (1.326–2.204)	
**Are you a conventional cigarette smoker?**		
No	Ref	0.089
Yes	1.496 (1.018–2.197)	0.040
Ex-smoker	0.883 (0.513–1.520)	0.653

## Discussion

This study is the first to examine the use, knowledge, and perceptions of e-cigarettes among university students in Jordan. Awareness of e-cigarettes is high among young adults, almost all participants (97%) reporting to have heard of e-cigarettes. However, the prevalence of e-cigarette use is low with only around 10% of smokers use e-cigarette reflecting low popularity among university students. Further, around 50% of the participants perceived e-cigarettes as less harmful and less addictive than conventional cigarettes. Findings from the current study confirm that tobacco users among university students underestimate the harmful risks from smoking either conventional cigarettes or e-cigarettes. The use of e-cigarettes has increased globally. In 2018, a local “Put it Out” campaign was launched to enforce the Jordanian public health law number 47, which prohibits cigarette smoking in public areas including universities [29].

Research outputs revealed that the prevalence of conventional cigarette smoking among the studied cohort was (11.3%) and that of e-cigarettes was (10.5%) with approximately 60% of daily use. In the current study, the reported number of conventional cigarette users was lower than the previously reported estimates (25.9% -29%) between 2011–2012 [30, 31]. This could be attributed to the lower proportion of males in the current study (29.8%) compared to previous studies (51%– 37%) [30, 31]. In Jordan, females’ tobacco smoking is not fully considered as a culturally acceptable behavior.

The reported prevalence of e-cigarette users in this study is higher than that reported among medical students from other countries such as Poland (3.5%) [32], and lower than that reported from USA (24.2%) [26] and Malaysia (40.3%) [33]. Despite the common culture between Jordan and Saudi Arabia, percentage of Saudi university students using e-cigarettes was higher (27.7%) [3]. This could be due to the difference in socio-economic status between students. The use is expected to be higher among those with higher socio-economic status whom are able to afford such expensive e-cigarette starter kit in products and equipment, where your adults needs to invest a larger first time sum for purchasing in comparison to conventional cigarette. The behavioral economics literature has shown with rising prices will likely lead to reduction in e-cigarette sales, where just as in cigarette sales, they are sensitive to price changes [34–36]. Studies showed that the level of consumption of cigarettes and e-cigarettes are not independent, where most current e-cigarette users are either current or former smokers [37–39]. Thus, suggesting that changes in e-cigarettes pricing may potentially impact both vaping and smoking behaviors. These findings as well as variations in tax on tobacco products between countries and differences in the percentage of smokers could potentially be important factors affecting the use and prevalence of e-cigarettes.

A quarter of respondents (26.5%) reported that their intent to quit conventional cigarette smoking was the reason to use e-cigarettes. Similar findings were reported from Polish and American students [26, 32]. Consistent with previous researches [3, 33], there was a substantial overlap between conventional cigarette and e-cigarette use. In the present study, dual smoking of e-cigarettes and conventional cigarettes was reported by 32.1% of the participants. These findings suggest e-cigarettes use may worsen rather than resolve problematic behavior of tobacco use among young people. The second reported reason to use e-cigarette was curiosity (22%) which might be influenced by the attractive marketing efforts of e-cigarettes manufacturers. More than 20% of respondents believed that e-cigarettes are safer than conventional products. Such finding is concerning, as an association between the current use of conventional cigarettes and potential future use of e-cigarettes has been demonstrated in previous research [40].

Regarding the reported sources of information about e-cigarettes, our study found that social media was the most common source followed by friends and family members. These informal sources of information can contribute to many misconceptions identified among participants. Similar findings were reported in previous studies [41, 42]. It is notable that social media has become an essential part of almost every university student’s life. Research studies have highlighted the possibility of a significant association between exposure to e-cigarette marketing with increased likelihood and susceptibility to e-cigarettes use among youth [14, 15, 43].

Marketers are aware of the appealing social media to young people, as unregulated advertising and promotions of e-cigarettes are heavily available on social networks such as Twitter, Facebook and YouTube [44–46]. Although there is no evidence that has approved e-cigarettes as smoking cessation aids, and their safety profile has not been proven, advertisements often portray opposite messages. In fact, it is difficult to control or regulate the quality of information shared by marketing sources on social media platforms [47, 48]. Such results highlight the necessity of developing national interventional programs to increase university students’ awareness about e-cigarettes harms.

Several studies showed that users of conventional cigarettes are more likely to use e-cigarettes [49, 50], hence the need to better understand the external factors and their influence on perceptions about e-cigarettes is crucial. This was reflected from our perception items that assessed perceived health and behavioral outcome expectations as related to conventional cigarette use. In general, this study showed a significant association between conventional cigarette smokers and their perception on e-cigarettes being less harmful, less addictive, and less dangerous than conventional products. Nearly 62% of the participants would advise friends or family members to smoke e-cigarettes instead of conventional cigarettes. The same perception of considering e-cigarettes less harmful than conventional cigarettes was found in a study among general population in the United Kingdom [51]. Since most of the participants in this study (63.7%) were from medical college, as future health professionals, it is important to educate students through training sessions and to guide them towards the use of evidence-based resources.

The current study demonstrates the differences in overall knowledge of medical and non-medical students. The harmful health outcomes and addictive effect of e-cigarettes appear to be better comprehended among medical students. These findings suggest that the knowledge acquired from the curriculum of medical colleges may enhance the awareness of the risk of e-cigarettes use. Nevertheless, a third of the students still showed misconceptions that e-cigarettes contain natural substances and they do not consider it as a source of second-hand exposure to nicotine. Likewise, previous study showed that many university students at one medical school at USA believed that alternative tobacco products such as e-cigarettes resulted in less diseases states as gastrointestinal, respiratory and cardiovascular diseases compared to conventional cigarettes [50]. Regardless the fact our study showed the medical students had statistically higher knowledge scores than those from non-medical programs, the mean scores from both groups was below 50% correct, suggesting illiteracy about e-cigarettes even among the most knowledgeable university students. Numerous studies in USA, Lebanon, Saudi Arabia and Thailand were consistent with our findings [3, 20, 21, 26, 52]. Thus, the needs to approach and communicate to college students in both medical and non-medical schools that any tobacco use behavior is harmful, regardless of the used tobacco products (e-cigarettes, cigarettes, and waterpipe), especially given the fact that e-cigarettes are found to be more addictive than traditional cigarettes [12].

Further, the current study shows that knowledge about e-cigarettes is associated with current use of e-cigarettes and conventional cigarettes and that there is less knowledge of harms and addictiveness for e-cigarettes among current users. The same feedback was also recorded in the study at Thailand, where students with less knowledge about the harmful effects of e-cigarettes were found more likely to use them [52]. Many healthcare professionals may encounter e-cigarette users [53, 54], and thus should be educated on e-cigarettes through their educational curricula and awareness campaigns to prepare medical students for better clinical decision making and patient counseling with regards to smoking and e-cigarettes.

This study provides the most comprehensive evaluation to date of e-cigarettes use, perception and knowledge among university students in Jordan. It is characterized by a relatively large sample size of (n = 1259) and including respondents from many public and private universities, representing different geographical areas supporting a good generalizability of our results to the general population of university students in Jordan. However, this study has limitations. First, data was self-reported and subjected to recall and social desirability biases. Second, participation was susceptible to self-selection or volunteer bias, which could lead to under and/or over-estimation of our estimates.

In conclusion, the use of e-cigarettes in Jordan is less popular compared to other countries. The misconception that e-cigarettes help with conventional smoking cessation was the most commonly reported reason for its use among study participants. Educational interventions are needed to resolve the widespread misconceptions about e-cigarettes; as being less harmful and less addictive among university students. The overall knowledge among university students were low, the average scores were less than 50% correct, suggesting gap of knowledge about e-cigarettes even among the medical students, who are in need to more education as future healthcare professionals about e-cigarettes to effectively advise patients and the community at large. Such findings highlight the urgent needs for antismoking mass media campaigns, which can focus on social media portals campaigns as a promising e-cigarette and conventional cigarette educational strategy to reach youth college students, directly and potentially through peer-to-peer sharing.

## Supporting information

S1 Fig Sources of information about e-cigarettes. (TIF)

S2 Fig Reasons behind using e-cigarettes for the first time. (TIF)
