# Supplementary figures and images for: E-cigarettes use among university students in Jordan: Perception and related knowledge
Source: PLoS One. 2021 Dec 31;16(12):e0262090. doi: 10.1371/journal.pone.0262090 (PMC8719738; doi:10.1371/journal.pone.0262090)

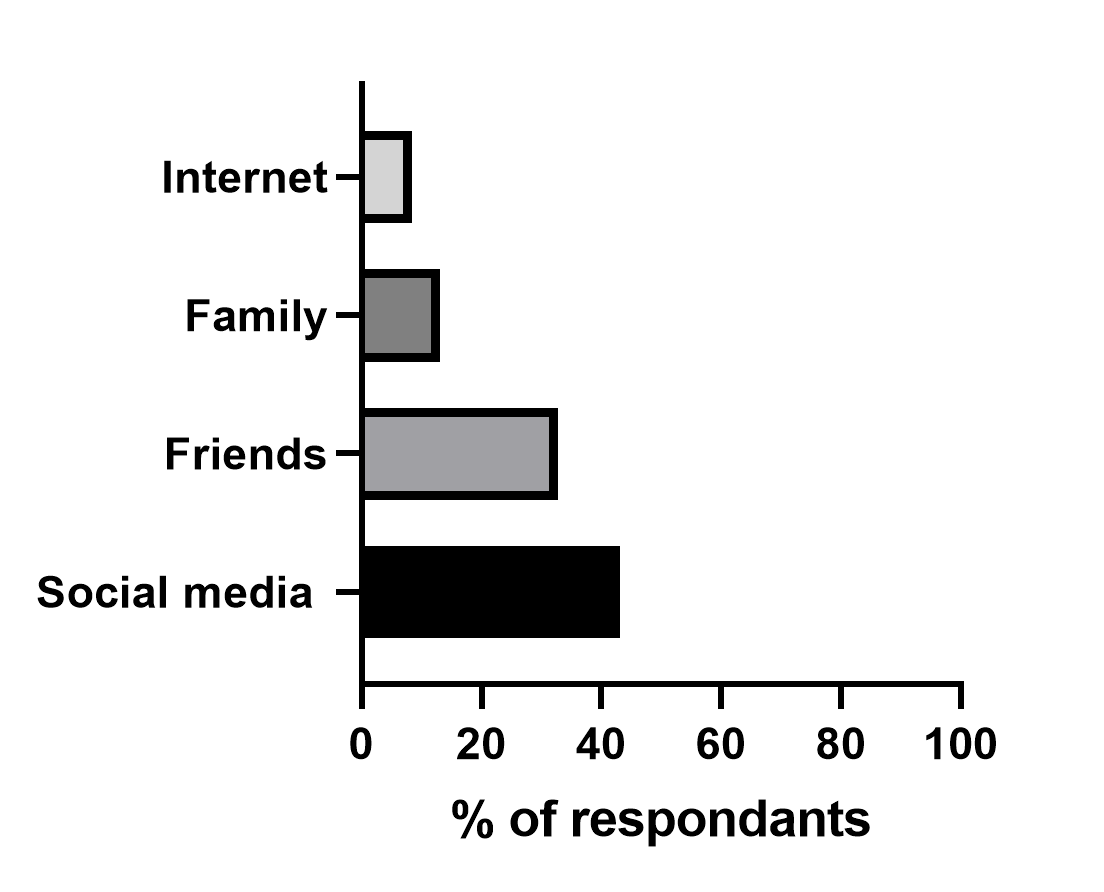

Supplement: S1 Fig — (TIF) [file pone.0262090.s001.tif]

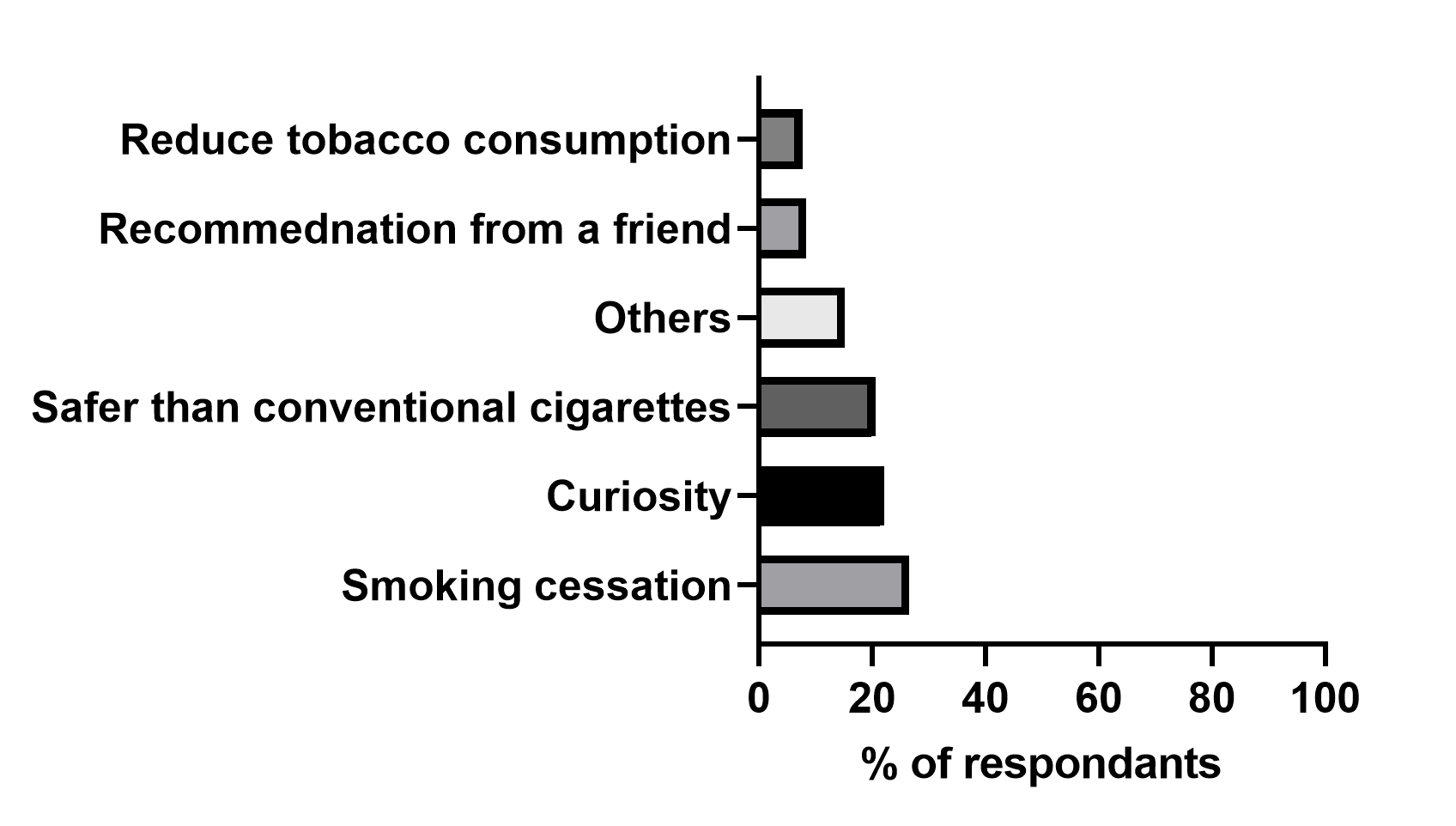

Supplement: S2 Fig — (TIF) [file pone.0262090.s002.tif]
